# Hsa_circ_0000994 Inhibits Pancreatic Cancer Progression by Clearing Immune-Related miR-27a and miR-27b

**DOI:** 10.1155/2022/7274794

**Published:** 2022-05-27

**Authors:** Jun Liu, Wei Yuan, Dongwei Gong

**Affiliations:** ^1^Department of Hepatobiliary Pancreatic Surgery, Hainan General Hospital, Hainan Affiliated of Hainan Medical University, Haikou, Hainan, China; ^2^Department of emergency surgery, Hainan General Hospital, Hainan Affiliated Hospital of Hainan Medical University, Haikou, Hainan, China

## Abstract

Pancreatic cancer (PC) is a common cause of cancer death. Although more and more evidences suggest that circular RNAs (circRNAs) are associated with the development of cancer, the biological function of circRNAs in PC has not been fully explored. Based on previous studies, Hsa_circ_0000994 was screened out, and its clinical significance, functional role, and mechanism in PC are poorly studied. In various cell lines, 50 PC tissues, and an equal number of normal tissues, RT-qPCR was used to identify expression level of Hsa_circ_0000994. The impact of Hsa_circ_0000994 on metastasis, cell proliferation, and apoptosis was detected using functional loss and functional gain tests. An animal study was also conducted. Underlying mechanisms of Hsa_circ_0000994 were revealed by luciferase reporter gene detection. Hsa_circ_0000994 was lowly expressed in PC tissues as well as various PC cell lines, and this low expression was closely related to cancer. In terms of functional testing, Hsa_circ_0000994 suppressed core ability of PC cells, including proliferation, migration, and invasion ability. Xenotransplantation studies further confirmed the effect of Hsa_circ_0000994 in promoting cell growth. Mechanically, Hsa_circ_0000994 inhibited miR-27a and miR-27b. Hsa_circ_0000994 inhibited the cancer cells through the effect on miR-27a and miR-27b. In summary, a circRNA with tumor suppressor effects on PC has been elucidated.

## 1. Introduction

Pancreatic cancer (PC) is a rare but highly malignant gastrointestinal tumor. Its morbidity and mortality have been rising in recent years and become a common serious cancer in the world [1]. Though advances have been made in clinical technology, more than 95% of patients with PC died within 5 years [2]. PC progression is a multistep and complex process involving activation or silencing of various oncogenes. Therefore, studying the molecular mechanism of PC occurrence is important for finding potential therapeutic options.

Circular RNA (circRNA) is a noncoding RNA, which has a 5′-end cap and a 3′-end polyA tail [3]. The circRNA is widely distributed in genomic regions of organisms, with more than 80% of their target coding genes [4]. Because high-throughput sequencing technology has been developing in recent years, discovery of many highly stable and conserved circRNAs in the human genome has improved the study of circRNA [5, 6]. New research demonstrates that through sponge microRNA (miRNA), circRNA has the ability of gene regulation [7]. CircRNA also has a great impact on tumorigenesis. For example, Hsa_circ_0000977 drives the PC process through sponge miR‐874‐3p and regulates PLK1 [8]. Other noncoding RNAs have also been discovered to have a crucial regulatory function in cancer. The miR-29c-3p/KIF4A axis could act as a target for ovarian cancer patients. The LATS2 is targeted by miR-92, and si-LATS2 led to enhanced YAP1 translocation and PD-L1 upregulation. Hence, the above evidence supports the importance of exploring the functional role of circRNA in PC.

We first found the regulatory potential of Hsa_circ_0000994 on PC. At the same time, the correlation between Hsa_circ_0000994/miR-27a and Hsa_circ_0000994/miR-27b was determined, and the regulatory effect of Hsa_circ_0000994 on miR-27a or miR-27b was further verified. Our study indicated the underlying therapeutic impact of Hsa_circ_0000994 in PC patients.

## 2. Materials and Methods

### 2.1. Sample Source

This study was approved by the Ethics Committee of Affiliated of Hainan Medical University. 50 pairs of fresh PCs and corresponding healthy tissues were obtained from Affiliated of Hainan Medical University. All patients obtained informed consent before the start of the study. All human PC cell lines include Capan‐2, AsPC‐1, BxPC3, PANC1, and SW1990. This cell line was isolated in 1978 from splenic metastasis of a stage II pancreatic cancer. The normal cell line HPDE was cultured in RPMI1640 or DMEM with additional 10% fetal bovine serum (FBS) and 1% penicillin and streptavidin, placed in a 37°C incubator with 5% carbon dioxide. In addition, we used the KM-plotter database to evaluate the prognostic information.

### 2.2. Cell Transfection and RT‐qPCR

We placed PC cells into serum-free medium and transfected it using siRNA which targeted to Hsa_circ_0000994 (si-circ). A nonspecific nucleotide served as a negative control (si-NC). In order to overexpress Hsa_circ_0000994, cloning of whole circular RNA and artificial inverted repeats into pcDNA3.1 (+) vector. The medium was changed after mingling with Lipofectamine 3000 (Invitrogen) for 6 hours. Transfected cells were then collected at a specified time point for further detection.

TRIzol solution (Invitrogen, Carlsbad, MA) was utilized for the extraction of total RNA. 25 *μ*L enzyme-free water was used to dissolve the extracted RNA. cDNA was obtained by reverse transcription of 1 *μ*g of the extracted RNA. Finally, the SYBR Green Mix kit (Takara, Otsu, Japan) was used for quantitative PCR analysis, with a total of 3 replicates and 35 cycles. U6 was utilized as internal controls for miRNAs, while GAPDH was used for mRNA, respectively. The 2^−ΔΔCt^ method was used to determine relative expression.

### 2.3. Luciferase Reporter Assay

PC cells were cotransfected with wild or mutant Hsa_circ_0000994 plasmid, miR-27a/miR-27b mimic, or negative control. The Dual‐Luciferase Reporter Assay System (Promega) was used to identify the luciferase intensity in 24 hours after transfection.

### 2.4. Detection of Cell Proliferation

Cell proliferation was assayed by CCK-8 (Dojindo, Japan) according to the instructions. Briefly, 1500 transfected PC cells were transplanted into a 96-well plate. CCK-8 solution was added from 0 to 96 h with the interval of 24 h. The incubation of cells was performed in dark area for 2 h, and a 450 nm wavelength was used to measure the absorbance.

6-well plates placed in a cell culture incubator were used to culture the transfected PC cells. After 12 days of staining, visible colonies were counted and photographed.

### 2.5. Tumor Formation Assays

1 × 10^7^ PANC1 cells transfected by shCtrl or sh-Hsa_circ_0000994 were subcutaneously injected into 8-week-old female BALB/C nude mice. There were 7 mice per group. Measurement of tumor weights was measured after the sacrifice in 15 days after inoculation. Afterwards, the expression of Ki67 was verified by immunohistochemistry (IHC).

### 2.6. Cell Apoptosis

Treated PC cells were digested and collected with binding buffer. Annexin V-FITC and propidium iodide (PI) were added and incubated for 15 minutes at room temperature. Stained cells were detected on a FACScan flow cytometer (BD Biosciences).

### 2.7. Transwell Assay

Transwell inserts were measured for invasive capacity using the Matrigel (BD Biosciences) method. The cells were suspended in the cell suspension; then, FBS (20%) in DMEM was added in the lower chamber. After 24 h, cells in the upper chamber were wiped with cotton swabs. The fixation and stain of these cells were performed using 100% ethanol and 0.5% crystal violet solution, respectively. The random field is photographed to determine cell number under the microscope.

### 2.8. Data Analysis

Prism 8 software (GraphPad Software) was used in all statistical analyses. The results are represented as mean ± SD. *P* values were compared using Student's *t* test or ANOVA, and statistical differences were *P* < 0.05.

## 3. Results

### 3.1. Downregulation of Hsa_circ_0000994 in PC

**Figure 1:**
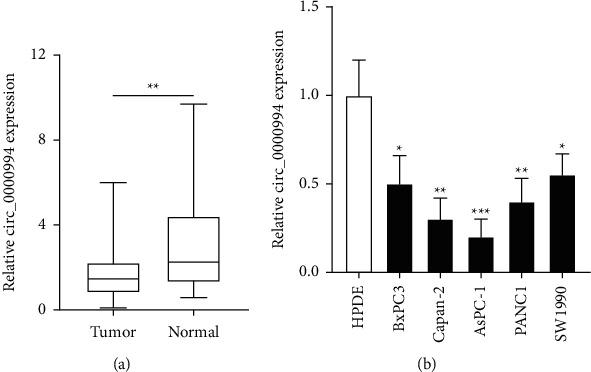
The expression levels of Hsa_circ_0000994 in PC tissues and cells. (a) RT-qPCR used to detect the relative expression of Hsa_circ_0000994 in PC tissues and normal tissues. (b) RT-qPCR detection of relative expression of Hsa_circ_0000994 in PC cells and HPDE.

In PC and normal tissues, RT-qPCR analysis was used to detect the expression regularity of Hsa_circ_0000994. Tumor tissue was associated with lower expression of Hsa_circ_0000994 compared to normal tissue (Figure 1(a)). Subsequently, the expression level of Hsa_circ_0000994 was identified in PC cells and HPDE. The data indicated that Hsa_circ_0000994 was significantly downregulated in all five PC cells (Figure 1(b)).

### 3.2. Silencing Hsa_circ_0000994 Promoted PC Cells Proliferation, Migration, and Invasion

**Figure 2:**
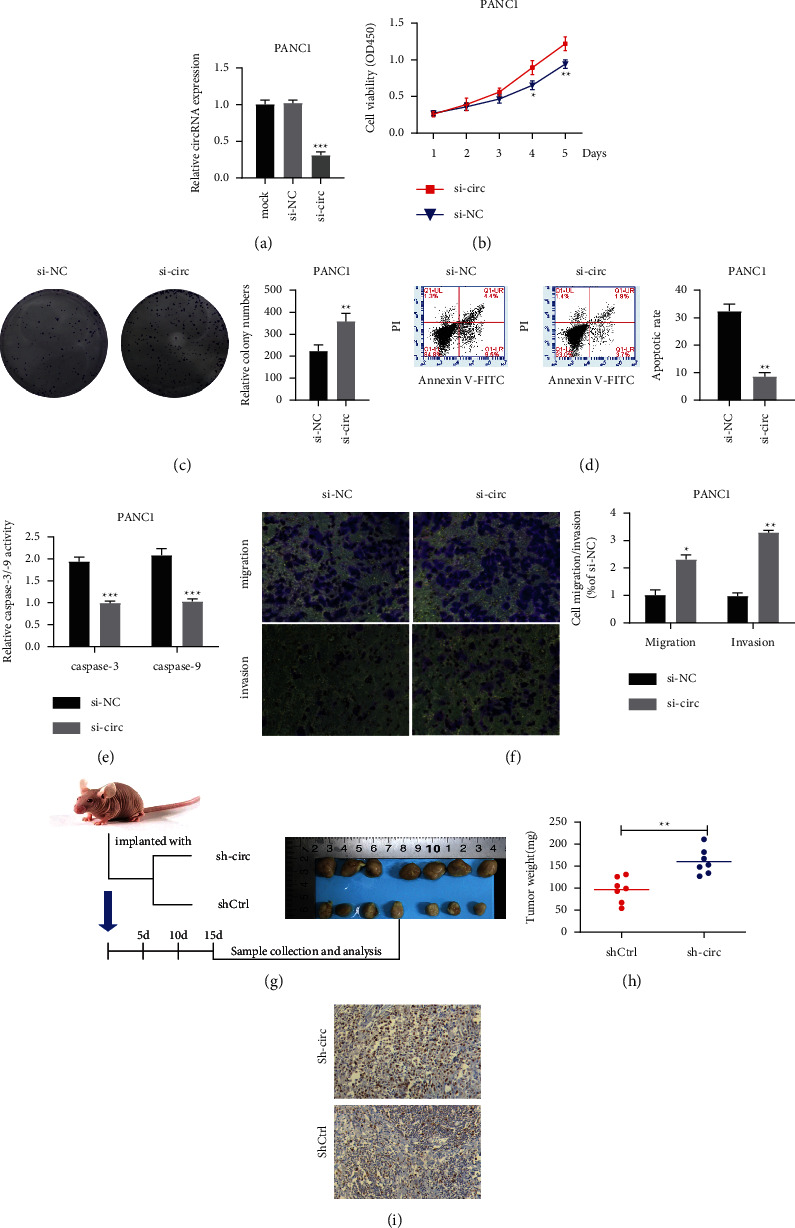
Silencing Hsa_circ_0000994 promoted carcinogenic properties of PANC1 cells. (a) RT-qPCR detection of the expression level of Hsa_circ_0000994 in PANC1 after transfection. (b) Detection of cell viability of transfected PANC1 by the CCK-8 method. (c) The clonogenic ability of PANC1 after transfection detected by clonogenic assay. (d) Apoptosis of PANC1 detected by flow cytometry. (e) Caspase-3/-9 activity in PANC1 after transfection. (f) The migration and invasion ability of transfected PANC1 cells detected by the transwell method. (g) Nude mice tumors after transfection of PANC1. (h) 15 days after injection, tumors were excised and weights were measured. (i) IHC detection of Ki67 expression and cell number. IHC, immunohistochemical. ^*∗∗∗*^, *P* < 0.001, ^*∗∗*^, *P* < 0.01, and ^*∗*^, *P* < 0.05. Overexpression of Hsa_circ_0000994 inhibits cell proliferation, migration, and invasion.

To determine the role of Hsa_circ_0000994 in PC, knockout studies were performed in PANC1 cells. 72 hours after transfection, the si-circ_0000994 group had significant knockdown expression (Figure 2(a)). Compared to the control group, improvement of growth and colony-forming was observed in knockdown expression of Hsa_circ_0000994 (Figures 2(b) and 2(c)). In addition, the promoting effect is also related to the inhibition of apoptosis caused by the knockout of Hsa_circ_0000994 gene (Figure 2(d)). The activity of Caspase-3/-9 was attenuated (Figure 2(e)). In terms of cell metastasis characteristics, knockdown of Hsa_circ_0000994 enhanced the migration and invasion abilities of PANC1 (Figure 2(f)). Further animal experiment was conducted to clarify the function of Hsa_circ_0000994 on inhibiting cell growth. Tumors were collected 15 days after injection (Figure 2(g)), which indicated the downregulation of I_circ_0000994 significantly increased cell growth, resulting in increased tumor weight (Figure 2(h)). In addition, the relative expression of Ki67 in tissues was detected by IHC staining. The number of Ki67 tumors in the sileIHsa_circ_0000994 was higher than that in shCtrl (Figure 2(i)), indicating strong cell proliferation. ^*∗∗∗*^, *P* < 0.001, ^*∗∗*^, *P* < 0.01, and ^*∗*^, *P* < 0.05.

**Figure 3:**
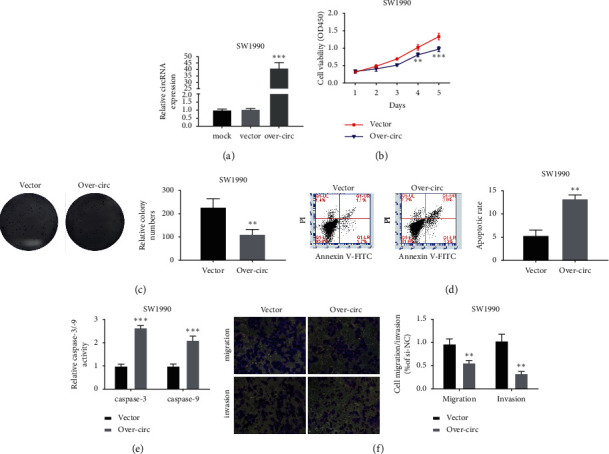
Overexpression of Hsa_circ_0000994 suppressed the carcinogenic properties of SW1990 cells. (a) RT-qPCR detection of the expression level of Hsa_circ_0000994 after transfection of SW1990. (b) Cell viability after transfection of SW1990 detected by the CCK-8 method. (c) Detection of clonogenicity after transfection of SW1990 by clonogenic assay. (d) Apoptosis of SW1990 detected by flow cytometry. (e) Caspase-3/-9 activity in SW1990 after transfection. (f) The migration and invasion ability of transfected SW1990 detected by the transwell method. ^*∗∗∗*^, *P* < 0.001, ^*∗∗*^, *P* < 0.01, and ^*∗*^, *P* < 0.05. Hsa_circ_0000994 bound directly to miR-27a/miR-27b in PC cells.

Then, we elucidated the role of Hsa_circ_0000994 by overexpression. Hsa_circ_0000994 was overexpressed in SW1990 cells (Figure 3(a)). Cell proliferation assays indicated that the ectopic expression of Hsa_circ_0000994 inhibited the growth and cloning ability of SW1990 (Figures 3(b) and 3(c)). Flow cytometry analysis indicated that overexpression of has_circ_0000994 increased the apoptosis rate (Figure 3(d)). The activities of Caspase-3/-9 increased in over-circ (Figure 3(e)). The metastatic characteristics were reduced after Hsa_circ_0000994 overexpression (Figure 3(f)). Overall, Hsa_circ_0000994 suppressed the malignant behavior of PC cells. ^*∗∗∗*^, *P* < 0.001, ^*∗∗*^, *P* < 0.01, and ^*∗*^, *P* < 0.05.

**Figure 4:**
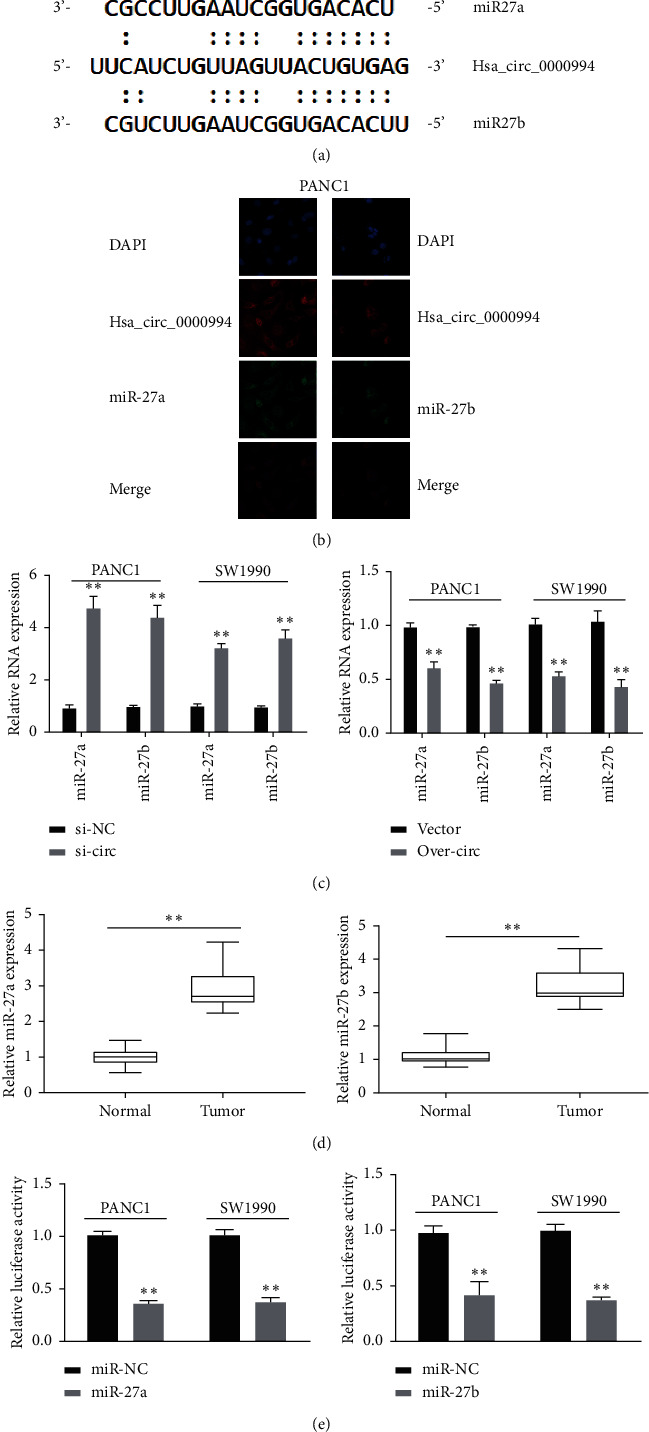
Hsa_circ_0000994 sponges miR-27a/-27b. (a) Schematic diagram of miR-27a/-27b binding sites in Hsa_circ_0000994. (b) Subcellular localization of Hsa_circ_0000994 and miR-27a/-27b detected by fluorescence in situ hybridization. (c) The expression of miR-27a/-27b in transfected PANC1 and SW1990 detected by RT-qPCR.(d) RT-qPCR detection of miR-27a/-27b expression in PC and normal tissue.(e) Luciferase reporter gene suggested that overexpression of miR-27a/-27b inhibited the activity of Hsa_circ_0000994 in PANC1 and SW1990. ^*∗∗*^, *P* < 0.01 and ^*∗*^, *P* < 0.05. The tumor suppressor function of Hsa_circ_0000994 depends on miR-27a and miR-27b.

Bioinformatics analysis predicts that miR-27a/-27b are potential targets of Hsa_circ_0000994. Potential binding sites for miR-27a/-27b are shown (Figure 4(a)). Interestingly, miR-27a and miR-27b were positively correlated with immune score, as confirmed by the ESTIMATE algorithm (Figure S1). Consistently, FISH testing confirmed that Hsa_circ_0000994 colocalized with miR-27a/-27b in the cytoplasm of PC cells (Figure 4(b)). Subsequently, we downregulated and overexpressed Hsa_circ_0000994 and detected changes in miR-27a/-27b. We found Hsa_circ_0000994 inhibited expressions of SW1990 (Figure 4(c)). Moreover, PC tumor expressed more miR-27a/-27b than normal tissues, while the expression level was opposite between miR-27a/-27b and Hsa_circ_0000994 (Figure 4(d)). The luciferase reporter gene test confirmed that Hsa_circ_0000994 was associated with miR-27a/-27b. The results showed that upregulation of miR-27a/-27b could significantly inhibit the activity of Hsa_circ_0000994 in PANC1 and SW1990 (Figure 4(e)). These results indicated that Hsa_circ_0000994 sponges miR-27a/-27b in PC. ^*∗∗∗*^, *P* < 0.001, ^*∗∗*^, *P* < 0.01, and ^*∗*^, *P* < 0.05.

**Figure 5:**
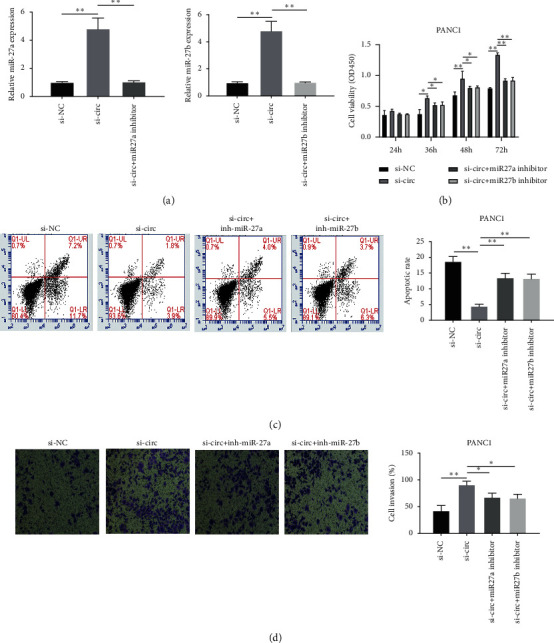
The anticancer function of Hsa_circ_0000994 depends on its regulation of miR-27a/-27b. (a) RT-qPCR detection of miR-27a/-27b expression in PANC1. (b–d) Detection of cell viability, apoptosis, and invasion ability in transfected PANC1. ^*∗∗*^, *P* < 0.01 and ^*∗*^, *P* < 0.05.

To explore whether the anticancer function of Hsa_circ_0000994 in PC is related to miR-27a/-27b, we inhibited miR-27a/-27b in PANC1 cells by transfection with the specific miR‐inhibitor (Figure 5(a)). Rescue experiments were then performed, and CCK-8, flow cytometry, and transwell invasion assay confirmed that si-Hsa_circ_0000994 promoted tumor growth, but this ability can be reversed by inhibiting miR-27a/-27b. In addition, cotransfection of si-Hsa_circ_0000994 with inh-miR-27a or inh-miR-27b reversed the effects of si-Hsa_circ_0000994 (Figures 5(b)–5(d)). Our study suggests that Hsa_circ_0000994 exerts an anticancer effect by sponging miR-27a/-27b in PC. ^*∗∗∗*^, *P* < 0.001, ^*∗∗*^, *P* < 0.01, and ^*∗*^, *P* < 0.05.

## 4. Discussion

PC is a rare but highly malignant tumor. Over the past few decades, people have made great efforts in the diagnosis and treatment of PC [9]. CircRNA is a stable and competitive novel endogenous RNA [10]. Previous studies have indicated the effects of circRNA on tumor cell regulation, indicating its potential value in cancer diagnosis and management [11]. In recent years, various circRNAs and miRNAs have been found in PC, thus providing a way to further explore their functions [12]. Several circRNAs have been found to be associated with the development of cardiovascular diseases, cancer, and neurological diseases. For PC, a previous study found that knockdown of Hsa_circ_0000977 may inhibit tumor development and progression [8]. In addition, Chen et al. reported the regulation function of circRNA_100782 in the growth of PDAC cells [13]. In consistent with their studies, our study found Hsa_circ_0000994 was associated with the development of PC and has a sponge effect on miR-27a/-27b. As far as we know, we first report the effect and potential mechanism of Hsa_circ_0000994 in PC.

CircRNAs were discovered a long time ago. However, their biological function has only been demonstrated in recent years. To identify the effect of Hsa_circ_0000994 in PC, we carried out a series of functional tests to clarify that Hsa_circ_0000994 has an important suppression effect on tumor cell. Considering that the potential correlation between PC and expression level of Hsa_circ_0000994, we detected the altered cell proliferation and metastasis characteristics of Hsa_circ_0000994 through functional loss and functional gain. In this study, the reduction of Hsa_circ_0000994 leads to downregulation of cell apoptosis and upregulation of cell proliferation. On the contrary, in SW1990, overexpression of Hsa_circ_0000994 can effectively inhibit cell proliferation and promote cell apoptosis. Further in vivo studies have also demonstrated the tumor suppressor function of Hsa_circ_0000994. We revealed that Hsa_circ_0000994 in PC can promote cell apoptosis through the caspase pathway. Although SW1990 show strong migration ability, overexpression of Hsa_circ_0000994 can significantly eliminate these cells. Transwell invasion experiments also confirmed such results.

In terms of the mechanism, circRNAs can play its carcinogenic/tumor suppressive effect in malignant tumors by sponge miRNAs. For instance, downregulation of the circNFIB1 gene promoted PDAC lymphangiogenesis and lymph node metastasis. In the mechanism, circNFIB1 acted as a sponge for miR-486-5p and reversed the effect of miR-486-5p [14]. Our study confirmed that Hsa_circ_0000994 could interact with miR-27a/-27b through bioinformatics analysis and luciferase reporter gene analysis. Previous studies have shown that miR-27a/-27b are tumor-promoting factors. The miR-27a exerts oncogenic effects by targeting Spry2 to regulate the malignant biological behavior of PC cells [15]. The miR-27b was upregulated in glioma samples and cell lines. Downregulation of miR-27b can inhibit glioma proliferation, induce apoptosis, and inhibit glioma invasion [16]. These studies suggest that miR-27a/-27b have tumor-promoting effects in tumors. We also found enhanced expression of miR-27a/-27b in PC. Further rescue experiments confirmed that Hsa_circ_0000994 played a tumor suppressor effect by direct sponge phagocytosing miR-27a/-27b in PC. In short, the Hsa_circ_0000994/miR-27a/miR-27b regulatory axis may inhibit the canceration and development of PC.

In summary, this study found the downregulation of Hsa_circ_0000994 in PC samples for the first time, revealing the clinical value of Hsa_circ_0000994. In addition, our data also illustrate how Hsa_circ_0000994 can suppress cancer in this deadly disease. However, the limitations of our study also deserve attention, for example, the immune promoting after upregulation of miR-27a/miR-27b expression has not been validated in vitro.

## Data Availability

The data used to support the findings of this study are included within the article.

## Supplementary Materials

Supplementary Materials Figure S1. Correlation analysis between the expression of miR-27a/miR-27b and the immune score.
