# Supplementary figures and images for: Hsa_circ_0000994 Inhibits Pancreatic Cancer Progression by Clearing Immune-Related miR-27a and miR-27b
Source: J Oncol. 2022 May 27;2022:7274794. doi: 10.1155/2022/7274794 (PMC9166970; doi:10.1155/2022/7274794)

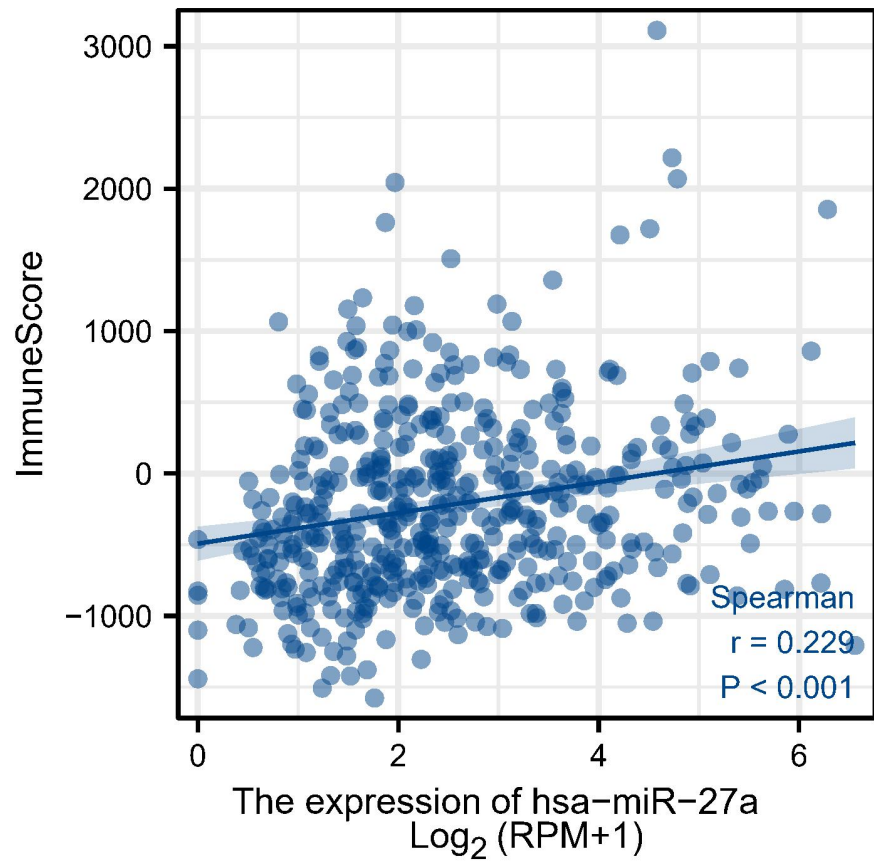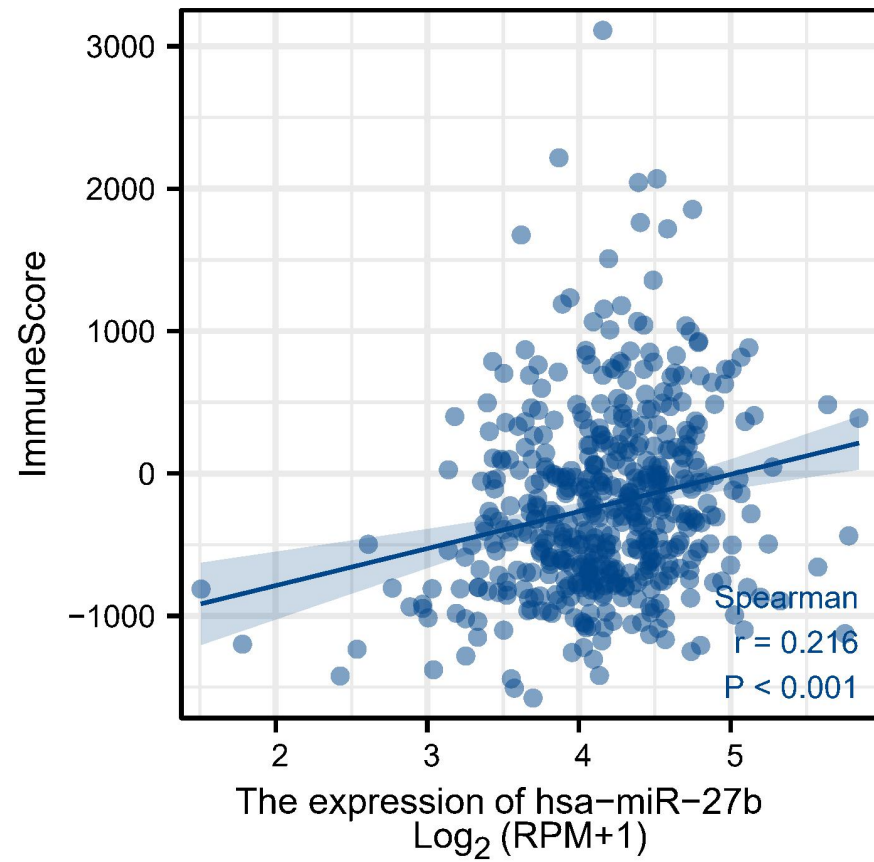

Supplement: Supplementary Materials — Figure S1. Correlation analysis between the expression of miR-27a/miR-27b and the immune score. [file 7274794.f1.pdf]
